# Pleural effusion and acute right heart failure due to a ruptured right sinus of Valsalva aneurysm and correction by surgical intervention

**DOI:** 10.34172/jcvtr.2020.25

**Published:** 2020-05-12

**Authors:** Jianmei Li, Qing Li, Yan Shen, Lihong Zhang, Chunmei Zhang, Tao Guo, Zihong Guo

**Affiliations:** Department of ICU, the Fuwai Yunnan Cardiovascular Hospital, Kunming, China

**Keywords:** Pleural Effusion, Acute Right-Sided Heart Failure, Sinus of Valsalva Aneurysm

## Abstract

In the study, we present the case of a 65-year-old male with rupture of right SVA into the right atrium that caused pleural effusion and acute right-sided heart failure (ARHF), which corrected by surgical intervention.

## Case Report

**Figure 1:**
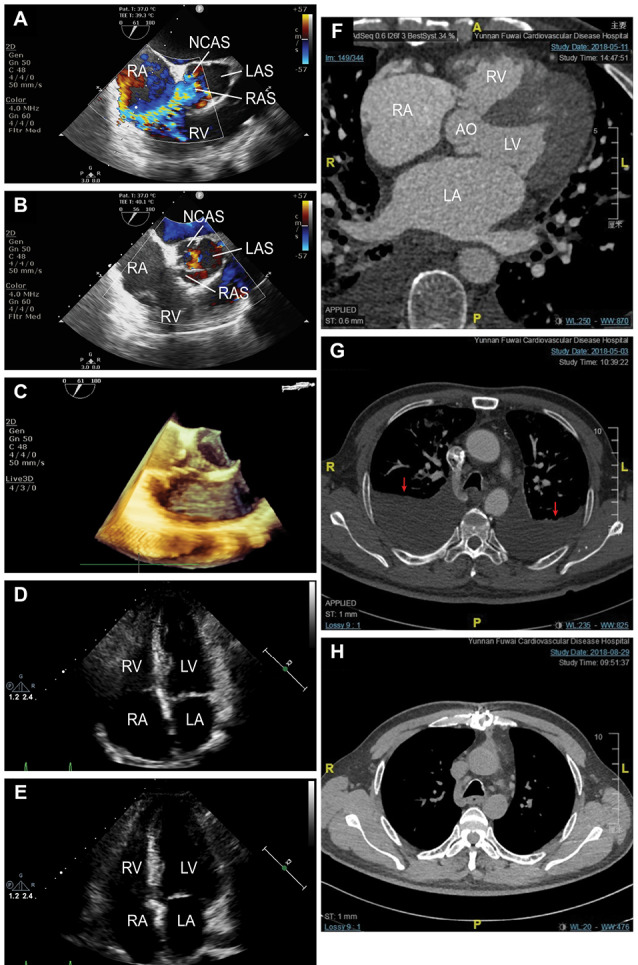
2D TEE continuous wave (CW) Doppler confirmed the presence of a ruptured sinus of Valsalva aneurysm (SVA) protruding into the right atrium (RA) (A) and corrected by surgical intervention (B). LAS, left aortic sinus; RAS, right aortic sinus; NCAS, noncoronary aortic sinus; LA, left atrium; LV, left ventricle; RA, right atrium; RV, right ventricle. 3D TEE confirmed a ruptured sinus of Valsalva aneurysm (SVA) shunt measuring 10.9 mm (C). The patient was further evaluated for coronary arterial anatomy preoperatively by coronary computed tomography angiography (D), which showed a large aneurysmal vascular structure protruding into the right atrium (RA).2D TTE showed a dilated RA (E) that was corrected 3 months postoperatively (F). CT findings. The CT scan taken on 3rd May 2018 shows a massive pleural effusion (arrows; G). A restaging CT scan shows pleural effusion in both lungs that disappeared after 3 months postoperatively (H).

We herein describe a rare case of a 65-year-old man who was admitted to our hospital for dyspnea and a gradual decline in functional capacity over the past 5 days in May 2018 with no other known cardiac history. Chest examination revealed dull percussion sounds and missing breathing sounds over his lower hemithorax. Cardiac auscultation revealed a grade 3/6 prolonged continuous cardiac murmur at the left sternal border over the first and second heart sounds. Jugular venous distention and hepatojugular reflux were present but without edema of the extremities, which indicated central edema rather than peripheral edema. Laboratory data revealed an NT-pro-BNP level of 2243.3 pg/mL. Infection-related indicators were negative, and the laboratory results suggested normal function of the liver and kidney. Echocardiography demonstrated that the right atrium (RA) and right ventricle (RV) were moderately dilated in apical four chamber view (RA: 53 × 42 mm preoperatively vs. 35× 32 postoperatively; RV: 30 mm preoperatively vs. 20 mm postoperatively) with moderate tricuspid regurgitation and a bicuspid aortic valve, and RV systolic function was mildly depressed, with a right ventricular fractional area change (RVFAC) of 35% preoperatively vs. 50% postoperatively. Thoracic radiographs demonstrated pulmonary congestion with both lower lobe infiltration and pleural effusion, which was refractory to diuretic therapy but was well controlled after thoracentesis, with a preoperative mean drained volume of 1020±268 ml (range 630–1400 mL, 10893 ml in total over 8 days) and a postoperative mean drained volume of 383±419 ml (range 15–1685 ml, 8193 ml in total over 18 days). After identifying the rupture, surgical correction under general anesthesia and hypothermal extracorporeal circulation was performed immediately. The successful procedure included surgical excision of the aneurysm under direct guidance of transesophageal echocardiography (TEE) (Figure 1 A-F), which displayed a lack of residual gradient or stenosis and showed an excellent rim of aortic tissue completely surrounding the aneurysm neck. The patient is doing well without recurrence of pleural effusion or ARHF at 3 months after surgery (Figure 1G-H).

## Discussion

To our knowledge, few cases of such a pleural effusion coexisting with ARHF have been reported. The natural mechanism of ARHF presenting with acute onset pleural effusion is not fully understood, and the treatment guidelines have not been established.

The clinical presentation of SVA varies from the compression effect of an unruptured aneurysm to acute heart failure in a ruptured aneurysm, which depends on the size of the aneurysm, the rapidity with which the aneurysm ruptures, and the underlying cardiac chamber into which it protrudes.in this case, the ARHF can occur because of abruptly increased RV preload and decreased RV contractility or increased pulmonary hypertension after rupture of SVA, which exacerbates RV dilation then drives a ventricular-interdependent effect limiting left ventricle (LV) filling, LV dysfunction aggravates further pleural effusion.^1^ This hypothesis can be partly demonstrated by no postoperative recurrence of pleural effusion with the disappearance of left-to-right heart flow during the 3-month follow-up.

Volume management during the preoperative period in cardiac surgery patients with ARHF and pleural effusion is not precisely understood. It is generally accepted to follow the guidelines for hemodynamic monitoring with central venous pressure (CVP) to determine intake and output.^1^ In this case, we determined that the CVP should not exceed the range of 8 to 12 mmHg to restore favorable intraventricular loading conditions and normalize interventricular interactions, which may induce more pleural effusion. Surgical repair of the aneurysm remains the ultimate treatment for ARHF-induced pleural effusion, while transcatheter closure devices can be used as surgical alternatives^2^. The choice of approach is determined by the presence of aortic valvular pathology such as aortic regurgitation, the size of the SVA, the presence of concomitant cardiac anomalies such as bicuspid aortic valves, which was implicated in this case.^2^ Intraoperative TEE was performed to monitor the procedure and to provide information regarding the involved sinuses, protrusion, and associated shunt or coexisting cardiac abnormalities.^3^ Ultimately, randomized trial data will be required for optimizing the management of these rare cases.

## Conclusion

SVAs are rare congenital or acquired cardiac defects that have been increasingly diagnosed as a result of improved imaging techniques, and SVAs are treated with transcatheter or surgical methods. In this report, a case of pleural effusion and ARHF due to a ruptured right SVA that was corrected by surgical intervention was presented.

## Competing interests

None.

## Ethical approval

The authors have no ethical conflicts to disclose. Informed consent was obtained from the patient.

## Funding

This work was supported by the Fuwai Yunnan Cardiovascular Hospital, Faculty of the Department of ICU.

## References

[1] Konstam MA, Kiernan MS, Bernstein D (2018). Evaluation and management of right-sided heart failure: a scientific statement from the American Heart Association. Circulation.

[2] Weinreich M, Yu PJ, Trost B (2015). Sinus of valsalva aneurysms: review of the literature and an update on management. Clin Cardiol.

[3] Afshar A H, Kolesnikov S, Pourafkari L (2015). Right Valsalva Sinus Aneurysm Protruding Into the Right Ventricle: A Case Report. J Cardiovasc Thorac Res.

